# *SLC38A1* and *STX11* are mitochondria-related biomarkers associated with immune infiltration in osteoarthritis

**DOI:** 10.3389/fgene.2025.1585775

**Published:** 2025-07-30

**Authors:** Wenxue Lv, Mingxiu Yu, Wenhai Yan, Yuli Cai, Weiguo Wang

**Affiliations:** ^1^ Department of Orthopedics, Affiliated Hospital of Shandong University of Traditional Chinese Medicine, Jinan, Shandong, China; ^2^ The First School of Clinical Medicine, Shandong University of Traditional Chinese Medicine, Jinan, China; ^3^ Department of Ultrasound Medicine, Affiliated Hospital of Shandong University of Traditional Chinese Medicine, Jinan, Shandong, China

**Keywords:** osteoarthritis, mitochondrial dynamics, mitophagy, immune infiltration analysis, single-cell analysis

## Abstract

**Background:**

Mitochondrial dynamics and mitophagy play crucial roles in osteoarthritis (OA); however, the specific contributions of mitochondrial dynamics-related genes (MD-RGs) and mitophagy-related genes (MP-RGs) remain unclear. This study aimed to elucidate the precise mechanisms linking these genes in the context of OA.

**Methods:**

OA-related transcriptome datasets and single-cell RNA sequencing (scRNA-seq) dataset incorporating MD-RGs and MP-RGs were utilized in this study. Hub genes were identified through differential expression analysis, weighted gene co-expression network analysis (WGCNA), and machine learning. A nomogram was then constructed based on the hub genes. Enrichment and immune infiltration analyses were performed on the hub genes, and key cell types were identified based on hub gene expression. Finally, the expression of the hub genes was validated using reverse transcription-quantitative polymerase chain reaction (RT-qPCR).

**Results:**

*SLC38A1* and *STX11* were identified as hub genes linked to mitochondrial dynamics and mitophagy in OA. These genes enabled the construction of a reliable nomogram for predicting OA risk. Enrichment analysis revealed that the top biological processes converged on the ECM–receptor interaction, underscoring its critical role in OA pathogenesis. Immune infiltration analysis uncovered significant disparities in 10 immune cell types, including activated CD4 T cells and central memory CD4 T cells, between OA patients and healthy controls. The levels of these immune cells were strongly correlated with the expression of *SLC38A1* and *STX11*. Additionally, endothelial cells, monocytes, and T cells emerged as key cellular players in OA. RT-qPCR validation showed that *SLC38A1* was significantly downregulated in OA samples, and *STX11* exhibited a similar trend, suggesting their potential roles in OA progression.

**Conclusion:**

This study identified *SLC38A1* and *STX11* as key genes linked to mitochondrial dynamics and mitophagy in OA. These findings provide a theoretical basis and valuable reference for the diagnosis and treatment of OA.

## 1 Introduction

Osteoarthritis (OA) is a chronic joint disease characterized by degenerative changes in the articular cartilage, synovium, and subchondral bone (Steinmetz et al., 2023). It is the most common joint disorder, with a prevalence that increases with age, affecting approximately 595 million people worldwide in 2020 (Steinmetz et al., 2023). OA has a series of complex pathological changes, including articular cartilage abrasion, synovial inflammation, subchondral bone remodeling, and osteophyte formation, which influence the development and progression of OA (Chu et al., 2020; Chilelli et al., 2024). OA is an irreversible degenerative disease, presenting with late-stage symptoms such as joint pain and deformity, which severely impact the daily activities of the patients (Duruöz et al., 2023). The causes of OA include age, gender, genetics, metabolism, and joint injury (Tibor and Ganz, 2022). Chondrocyte death, including autophagy, ferroptosis, apoptosis, and pyroptosis, contributes to the progression of OA (Guan et al., 2024; Zhu et al., 2023). Therefore, early diagnosis and timely intervention are extremely important as they can help delay disease progression and alleviate symptoms. In recent years, an increasing number of studies have confirmed that various dysregulated genes can serve as important diagnostic markers and therapeutic targets (Hadzic and Beier, 2023).

Mitochondria are essential organelles within cells that carry out and coordinate various metabolic processes and play a significant role in the development of OA (Blanco et al., 2004). Studies have shown that ERK1/2 is a key factor in promoting IL-1-induced mitochondrial fission and apoptosis in chondrocytes (Ansari et al., 2022). The mitochondrial network in normal chondrocytes remains intact, whereas several chondrocytes in OA cartilage exhibit excessive mitochondrial fragmentation (Blanco et al., 2004; Ansari et al., 2022). Mitochondrial dysfunction is also associated with OA (Blanco et al., 2004). Mitophagy is a process that selectively removes damaged or dysfunctional mitochondria through autophagy, thereby maintaining mitochondrial quality control and homeostasis. It has been reported that HIF-1α can alleviate cell apoptosis and senescence in chondrocytes under hypoxic conditions through mitophagy, thereby ameliorating cartilage degeneration in surgically induced OA mouse models (Hu et al., 2020). Additionally, curcumin exerts chondroprotective effects in osteoarthritis by promoting AMPK/PINK1/Parkin-mediated mitophagy (Jin et al., 2022). Therefore, genes related to mitochondrial dynamics and autophagy may serve as biomarkers for OA patients.

In summary, based on the single-cell and bulk transcriptome analyses, we have explored the potential of mitochondrial dynamics and autophagy as biomarkers for OA patients and their underlying molecular mechanisms, providing new insights for the early clinical diagnosis and treatment of OA patients.

## 2 Materials and methods

### 2.1 Data collection

OA-related transcriptome datasets (GSE57218 and GSE117999) and the single-cell RNA sequencing (scRNA-seq) dataset (GSE152805) (GPL20301) were downloaded from the Gene Expression Omnibus (GEO) database (http://www.ncbi.nlm.nih.gov/geo/). The GSE57218 dataset (GPL6947), containing 7 control and 33 OA cartilage tissue samples, was considered the training set (Ramos et al., 2014). The GSE117999 dataset (GPL20844), consisting of 12 OA and 12 control cartilage tissue samples, was considered the validation set. Detailed information on the samples in the training and validation sets is presented in Supplementary Table S1. The GSE152805 dataset included three OA cartilage tissue samples for scRNA-seq. A total of 23 mitochondrial dynamics-related genes (MD-RGs) were obtained from the published literature (Zhang et al., 2024). In total, 29 mitophagy-related genes (MP-RGs) were acquired from the Reactome database (Yang et al., 2022).

### 2.2 Single-cell analysis

Seurat objects from the scRNA-seq data were created using the Seurat package (version 4.1.0) (Tan et al., 2023). In the GSE152805 dataset, cells with fewer than 200 detected genes and genes expressed in fewer than three cells were filtered out. Subsequently, cells meeting the following criteria were retained: (1) the number of detected features (nFeature_RNA) was greater than 1,000 and less than 5,000; (2) total RNA counts (nCount_RNA) were below 30,000; and (3) the proportion of mitochondrial gene expression was less than 5%. Following data normalization using the NormalizeData function in the Seurat package, the FindVariableFeatures method was employed to select high-variable genes. Subsequently, principal component analysis (PCA) was performed, and the *p*-value of each principal component (PC) was calculated using the JackStraw function in the Seurat package. The significance of PCs was assessed using the ScoreJackStraw function, and PCs with statistically significant differences (*p* < 0.05) were selected for subsequent analysis. The scree plot of PCs was drafted using the ElbowPlot function in the Seurat package. Subsequently, unsupervised cluster analysis of cells was performed using the FindNeighbors and FindClusters functions of the Seurat package to identify cell clusters with a resolution of 0.3. Furthermore, cell clusters were annotated using the SingleR package (version 1.831) (Zhang et al., 2022), with the HumanPrimaryCellAtlasData from the celldex package used as the reference gene set, and cell subpopulations were subsequently identified. Moreover, marker genes for the different cell subpopulations were identified using the FindAllMarkers function in the Seurat package, and the scRNA-seq of differentially expressed genes (scRNA-seq DEGs) were selected using the Wilcoxon test, with a threshold of |log_2_fold change (FC)| > 0.25.

### 2.3 Differential expression analysis

The limma package (version 3.54.0) (Li et al., 2022) was used to identify bulk differentially expressed genes (bulk DEGs) between the OA and control groups in the GSE57218 dataset, with conditions of adj.p < 0.05 (FDR correction was used) and |log_2_FC| > 0.5. A volcano map of bulk DEGs was generated using the ggplot2 package, and the top 10 up- and downregulated bulk DEGs (based on log_2_FC) were displayed in a heatmap, which was created using the ComplexHeatmap package (version 2.15.1) (Gu, 2022).

### 2.4 Weighted gene co-expression network analysis

The MD-RGs and MP-RGs scores of samples in the GSE57218 dataset were calculated using the single-sample gene set enrichment analysis (ssGSEA) algorithm in the GSVA package (version 1.42.0) (Hänzelmann et al., 2013). Then, the difference in the MD-RGs and MP-RGs ssGSEA scores was compared between the OA and control groups using the Wilcoxon test (p < 0.05). To identify genes related to the ssGSEA scores of MD-RGs and MP-RGs, weighted gene co-expression network analysis (WGCNA) was performed using the WGCNA package (version 1.70–3) (Langfelder and Horvath, 2008). First, samples in the GSE57218 dataset were clustered to determine whether any outlier samples needed to be removed. Subsequently, a soft threshold (β) selection was performed to construct a co-expression network by selecting a scale-free R^2^ value greater than 0.9 and a mean connectivity value close to 0, ensuring that the constructed network corresponded more closely to a scale-free topology. The dynamic tree cutting algorithm was employed to partition modules, with a minimum requirement of 300 genes per module. Finally, the ssGSEA scores were integrated with the WGCNA module eigengenes, and the Pearson correlation between the modules and the phenotypic traits (MD-RGs and MP-RGs) was calculated using the cor function (|cor| > 0.3; *p* < 0.05). Genes in key modules were identified as the key module genes.

### 2.5 Identification and analysis of candidate genes

Candidate genes were obtained by taking the intersection of scRNA-seq DEGs, bulk DEGs, and key module genes. The results were visualized using a Venn diagram, which was created using the VennDiagram package (version 1.7.1) (Chen and Boutros, 2011). Furthermore, biological functions and pathways involved with the candidate genes were explored by Gene Ontology (GO) and Kyoto Encyclopedia of Genes and Genomes (KEGG) enrichment analyses. The above enrichment analyses were processed using the clusterProfiler package (version 4.2.2) (Yu et al., 2012) with *p* < 0.05.

### 2.6 Identification of hub genes

Based on candidate genes, least absolute shrinkage and selection operator (LASSO) (conducted a 10-fold cross-validation) and XGBoost (the importance of genes is quantified using the gain indicator) machine-learning algorithms were applied using the glmnet package (version 4.1–2) (Shi et al., 2023) and XGBoost package (version 1.6.2.1) (Kui et al., 2022) to identify signature genes. Then, the intersections of signature genes identified using two machine-learning algorithms were identified as the candidate hub genes. Furthermore, the expression of candidate hub genes was compared between the OA and control groups using the Wilcoxon test (*p* < 0.05). The genes that showed significant differences between the OA and control groups, with consistent expression trends in the GSE57218 and GSE117999 datasets, were identified as hub genes.

### 2.7 Construction of the nomogram

According to hub genes, a nomogram was established to predict the risk of OA using the rms package (version 6.5.0) (Pan et al., 2021). The receiver operating characteristic (ROC) curve was generated using the pROC package (version 1.18.0) (Robin et al., 2011) to explore the ability of the nomogram to distinguish between OA and control samples. Decision curve analysis (DCA) was performed to further evaluate the efficacy of the nomogram.

### 2.8 Functional analysis of hub genes

According to the expression of hub genes, OA samples in the GSE57218 dataset were divided into high and low expression groups. Gene set enrichment analysis (GSEA) was performed to find pathways using the clusterProfiler package. The reference gene set was c2.cp.kegg.v7.5.1.symbols.gmt, and the enriched threshold was | normalized enrichment score (NES)| > 1, NOM P < 0.05, and q < 0.25. GeneMANIA (https://genemania.org/) was used to identify genes that were functionally related to hub genes, and a gene–gene interaction (GGI) network was constructed. To explore tissue-specific expression of hub genes, BioGPS (http://biogps.org/) was utilized to predict the expression of hub genes in various tissues (species: human).

### 2.9 Immune infiltration analysis

To explore the correlation of hub genes and immune cells, the enrichment scores of 28 types of immune cells in the training set were calculated using the ssGSEA algorithm (Bindea et al., 2013). Subsequently, the Wilcoxon test was employed to compare the differences between immune cell enrichment scores between the OA and control groups (p < 0.05). After that, correlations between immune cells and between immune cells and hub genes were analyzed using the corrplot package (version 0.92) (Liu et al., 2022).

### 2.10 Regulatory network and drug prediction analyses

MicroRNA (miRNA)-targeting hub genes were predicted using the miRDB (https://mirdb.org/) and TargetScan databases (https://www.targetscan.org/vert_80/). The intersections of miRNAs derived from two databases were selected as key miRNAs. Then, long non-coding RNAs (lncRNAs) targeting key miRNAs were obtained from the starBase database (http://starbase.sysu.edu.cn/). To find potential drugs for the treatment of OA, Comparative Toxicogenomics Database (CTD, http://ctdbase.org) analysis was carried out. The lncRNA–miRNA–mRNA regulatory network and the drug–hub gene network were constructed using Cytoscape software (version 3.8.2) (Yue et al., 2021).

### 2.11 Cell communication and pseudo-time analysis

Cell communication of cell subpopulations was evaluated using the CellChat package (version 1.6.1) (Fang et al., 2022). After creating the CellChat object, importing objects to the ligand–-receptor database (CellChatDB.human), and performing preprocessing, the cell communication network was inferred. A bubble diagram was drafted to show the communication probability of ligand–receptor pair regulation from some cell groups to other cell groups. To identify key cells in the GSE152805 dataset, the expression of hub genes across cells was analyzed, and cells exhibiting high and widespread expression of the hub genes were defined as key cells. Cell trajectory differentiation of key cells was analyzed using the monocle package (version 1.0.0) (Zhai et al., 2020). In addition, the expression of hub genes in key cells differentiation was explored. Subsequently, enrichment of transcription factors (TFs) was evaluated using single-cell regulatory network inference and clustering (SCENIC, https://github.com/aertslab/SCENIC) based on single-cell data.

### 2.12 Reverse transcription-quantitative polymerase chain reaction

To investigate the expression levels of hub genes, an RT-qPCR experiment was performed. Initially, total RNA was extracted from the tissue samples using TRIzol reagent (Ambion, Austin, USA), followed by determination of RNA concentration. Subsequently, cDNA synthesis was carried out via reverse transcription using the SweScript First-Strand cDNA Synthesis Kit (ServiceBio, Wuhan, China). Finally, quantitative analysis was performed using the Universal Blue SYBR Green qPCR Master Mix (ServiceBio, Wuhan, China), and the gene’s relative expression level was calculated using the 2^–△△Ct^ method, with GAPDH used as an internal reference. Additionally, the primer sequences for the relevant genes are shown in Supplementary Table S2.

### 2.13 Statistical analysis

**FIGURE 1:**
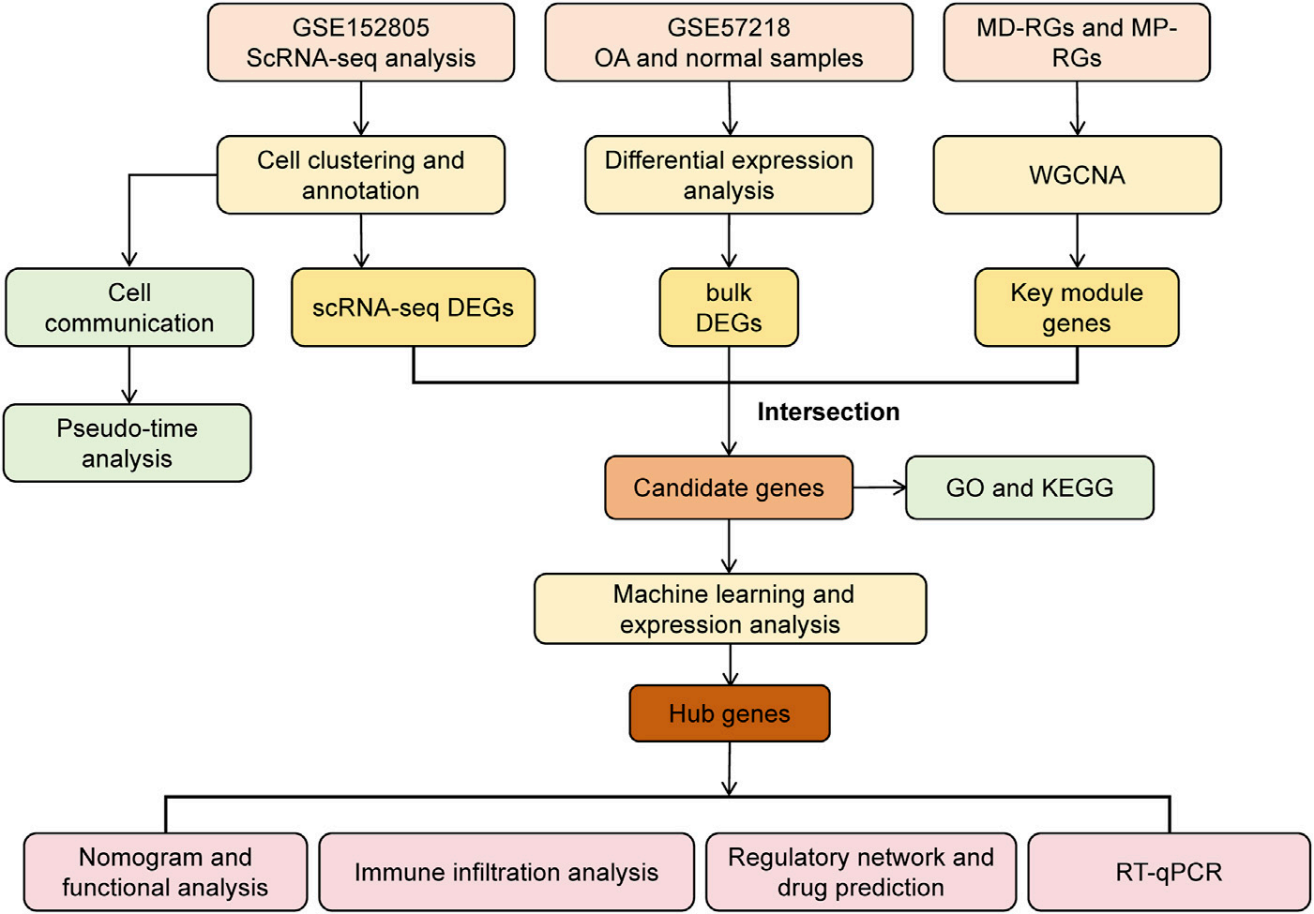
Analysis flowchart.

R software (version 4.2.2) was used to process and analyze data. Statistical significance between the two groups was assessed using the Wilcoxon rank-sum test. A *p*-value < 0.05 was considered statistically significant. The overall analysis process of this study is shown in Figure 1.

## 3 Results

### 3.1 Single-cell profiling revealed OA-related immune and endothelial subpopulations

**FIGURE 2:**
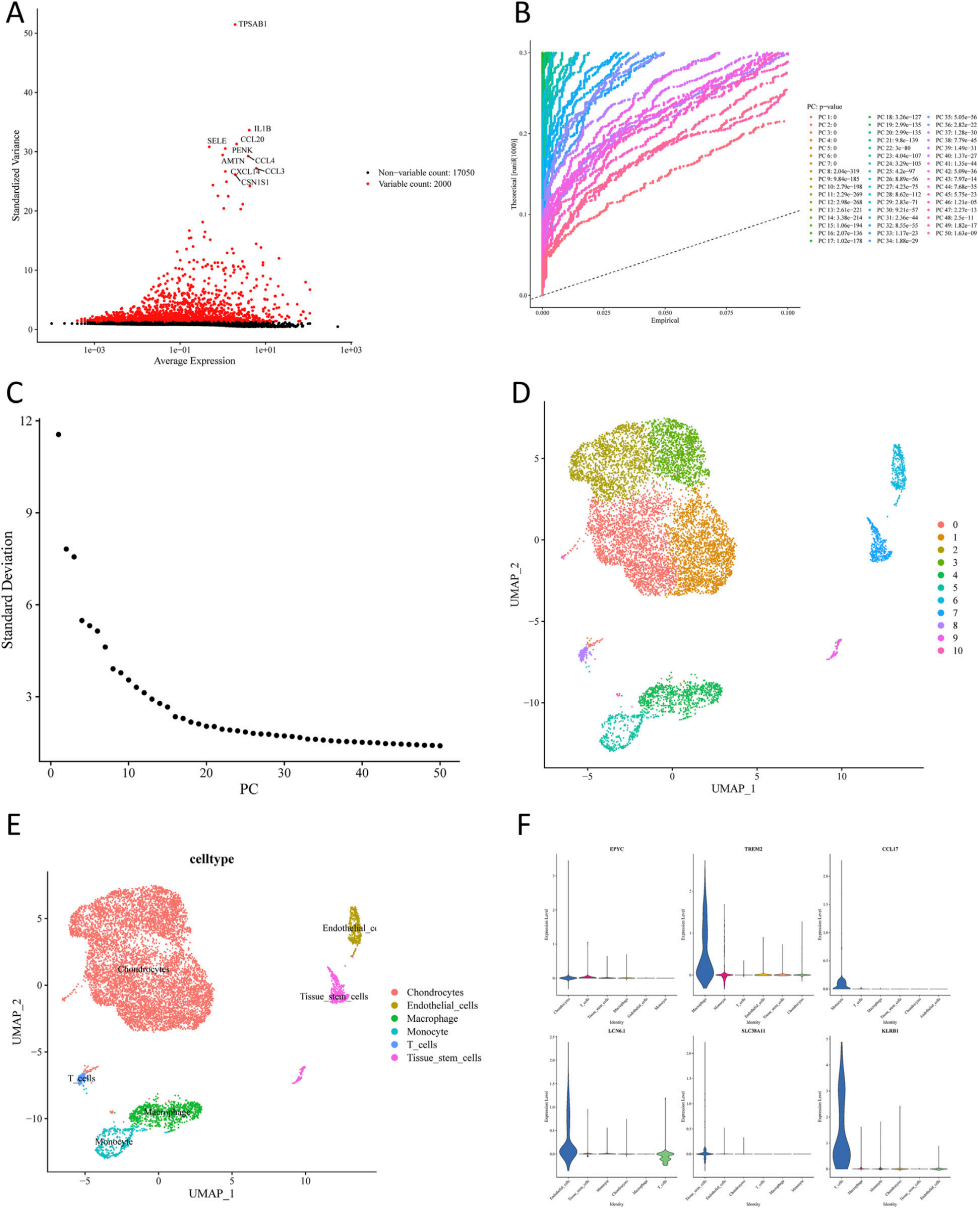
Single-cell sequencing analysis of osteoarthritis cartilage tissues. **(A)** Acquisition of highly variable genes. **(B)** Selection of meaningful PCs. **(C)** Principal component and standard deviation distribution plot. **(D)** UMAP plots of cartilage-associated cells colored by cluster. **(E)** Annotation of different cell clusters. **(F)** Expression levels of marker genes in various cell clusters.

After quality control of the scRNA-seq data from GSE152805, 10,032 cells and 19,050 genes remained following filtering (Supplementary Figure S1). Out of 2,000 highly variable genes, the p*-*value of the top 50 PCs was far less than 0.05. Then, the first 20 PCs were chosen for subsequent analysis through PCA (Figures 2A–C). As shown in Figure 2D, 11 cell clusters were obtained through uniform manifold approximation and projection (UMAP) cluster analysis. Subsequently, 6 cell subpopulations were annotated based on 11 cell clusters (chondrocytes, endothelial cells, macrophages, monocytes, T cells, and tissue stem cells) (Figure 2E). A total of 1,860 scRNA-seq DEGs were further selected among different cell subpopulations, and the expression of marker genes that relied on |log_2_FC| of each cell subpopulation was determined (Figure 2F).

### 3.2 Bulk DEGs associated with OA were screened out

**FIGURE 3:**
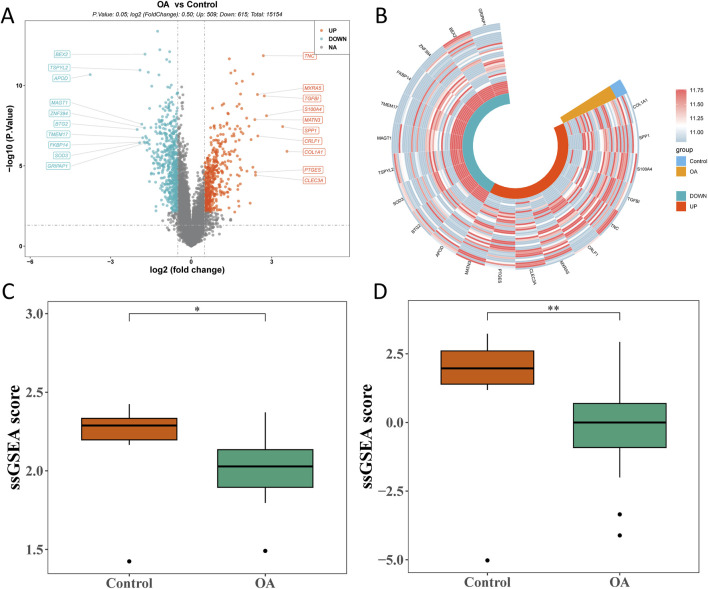
Bulk RNA sequencing analysis of osteoarthritis and control cartilage tissues. **(A)** Volcano plot of differentially expressed genes in bulk RNA-seq. **(B)** Circle heatmap of differentially expressed genes. **(C)** Comparison of mitochondrial dynamics_single-sample gene set enrichment analysis (MD_ssGSEA) scores between the OA and control groups. **(D)** Comparison of mitophagy_ssGSEA (MP_ssGSEA) scores between the OA and control groups. *p < 0.05 and **p < 0.01.

Through differential expression analysis, 1,124 bulk DEGs (509 upregulated and 615 downregulated genes) were screened out between the OA and control groups (Figures 3A, B). In the OA group, the ssGSEA score of MD-RGs and MP-RGs was significantly lower than that in the control groups (Figures 3C, D).

### 3.3 *SLC38A1* and *STX11* emerge as mitochondrial hub genes via integrative analysis

**FIGURE 4:**
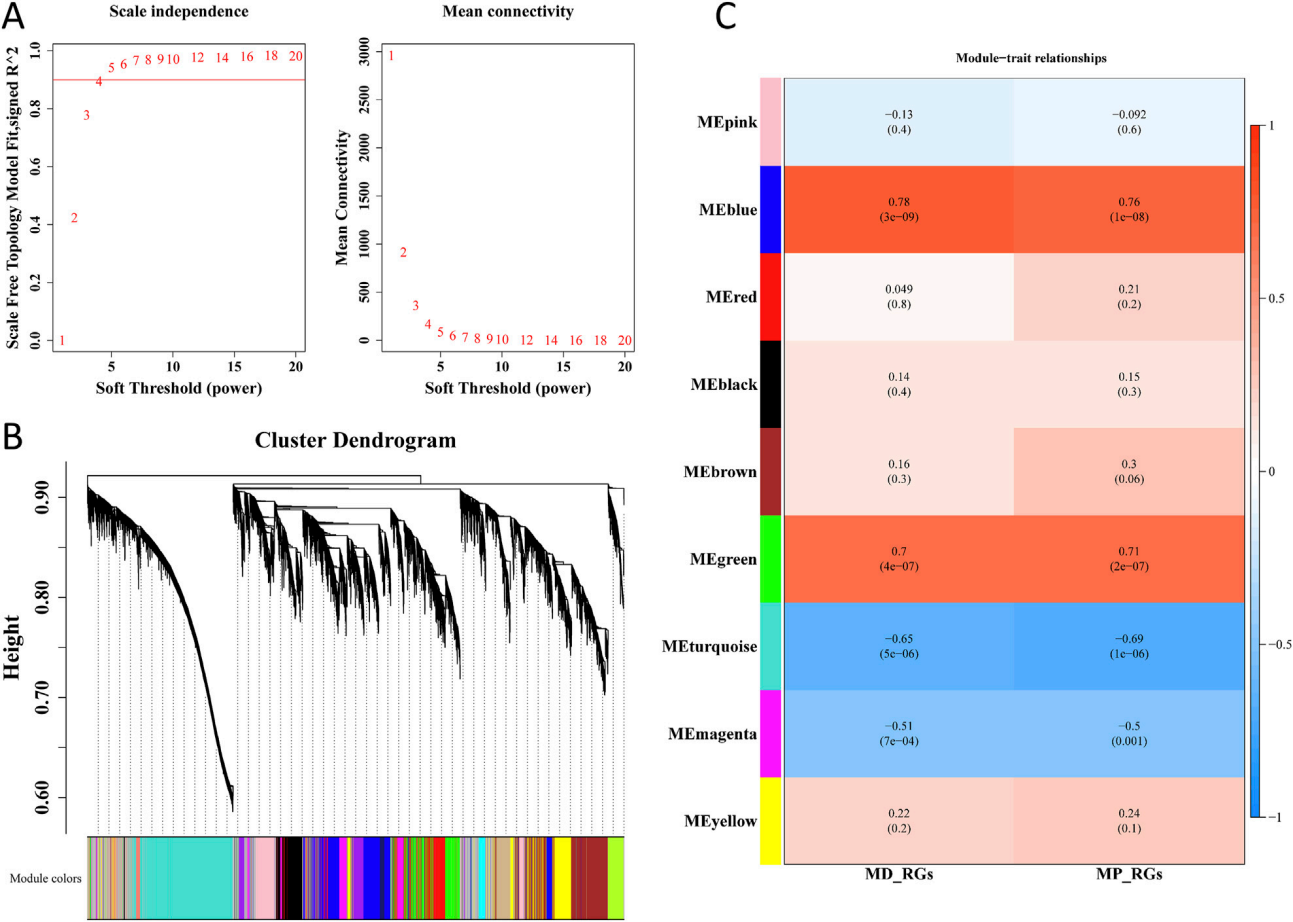
WGCNA. **(A)** Selection of the optimal soft threshold (β) value. **(B)** Dendrogram of gene clusters. **(C)** Correlation between modules and MD-RGs and MP-RGs.

Cluster analysis was performed on all samples in the GSE57218 dataset, and no outlier sample was found. Thus, all samples were used for subsequent analysis (Supplementary Figure S2). By setting R^2^ = 0.9 as the threshold, a soft threshold of 4 was selected, and data with mean connectivity close to 0 were filtered out (Figure 4A). Consequently, 9 co-expression modules were obtained through similarity analysis and by setting a minimum of 300 genes for each gene module (Figure 4B). Notably, as shown in Figure 4C, MEblue and MEturquoise modules, respectively, exhibited a strong positive (cor = 0.78 and 0.76; p < 0.05) and negative correlation (cor = −0.65 and −0.69, p < 0.05) with MD-RGs and MP-RGs ssGSEA scores, respectively. By combining 2,866 genes in the MEturquoise module and 2,420 genes in the MEblue module, 5,286 key module genes were finally acquired.

### 3.4 *SLC38A1* and *STX11* were selected as hub genes

**FIGURE 5:**
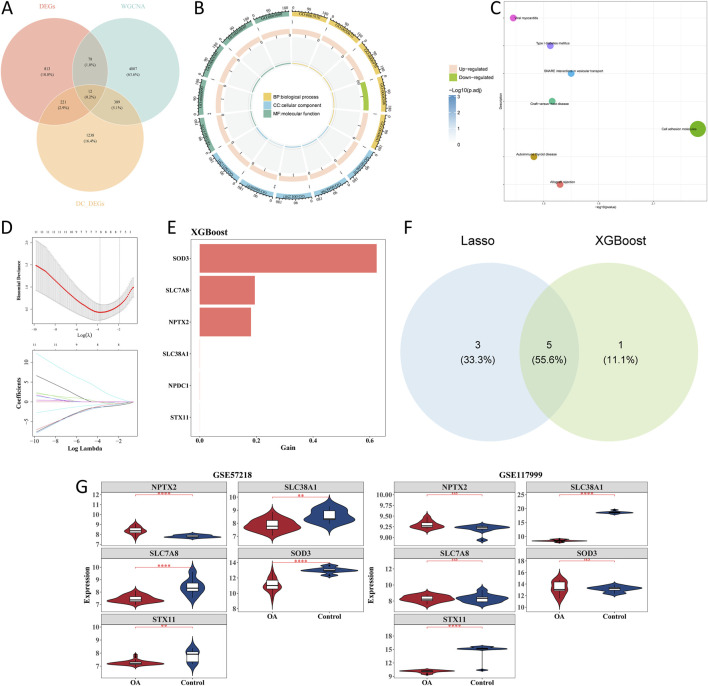
Screening of candidate genes with machine learning analysis. **(A)** Intersection of scRNA-seq differentially expressed genes (scRNA-seq DEGs), bulk DEGs, and key module genes. **(B)** GO enrichment of candidate genes. **(C)** KEGG pathway enrichment of candidate genes. **(D)** Lasso regression. **(E)** XGBoost analysis. **(F)** Intersection of candidate genes in Lasso and XGBoost analysis. **(G)** Violin plot of relative expression levels of candidate genes in GSE57218 and GSE117999 datasets. *p < 0.05, **p < 0.01, ***p < 0.001, and ****p < 0.0001.

Intersections of 1,860 scRNA-seq DEGs, 1,124 bulk DEGs, and 5,286 key module genes yielded 12 candidate genes (Figure 5A). Enrichment analysis of candidate genes showed that 236 GO terms and 7 KEGG pathways were enriched. Regulation of leukocyte-mediated cytotoxicity, solute: sodium symporter activity, and phagocytic vesicle membrane-related functions were GO terms significantly enriched by the candidate genes (Figure 5B). For KEGG pathways, viral myocarditis, type-I diabetes mellitus, SNARE interactions in vesicular transport, cell adhesion molecules, etc., were the main pathways (Figure 5C). In total, eight signature genes (*SLC7A8*, *SOD3*, *STX11*, *TSHZ1*, *NPTX2*, *SLC38A1*, *EFHD1*, and *ARID5B*) were selected by LASSO analysis when lambda was 0.0232298 (Figure 5D). Through XGBoost analysis, six signature genes, namely, *SOD3*, *SLC7A8*, *NPTX2*, *SLC38A1*, *NPDC1*, and *STX11*, were selected as gene features (Figure 5E). The intersection of eight signature genes in LASSO analysis and six signature genes in XGBoost analysis yielded five candidate hub genes, including *SLC7A8*, *SOD3*, and *STX11* (Figure 5F). Expression analysis of candidate hub genes showed that *SLC38A1* and *STX11* were obviously less expressed in the OA group in the GSE57218 and GSE117999 datasets (Figure 5G). Therefore, *SLC38A1* and *STX11* were identified as hub genes for further analysis.

### 3.5 Functional analysis of hub genes

**FIGURE 6:**
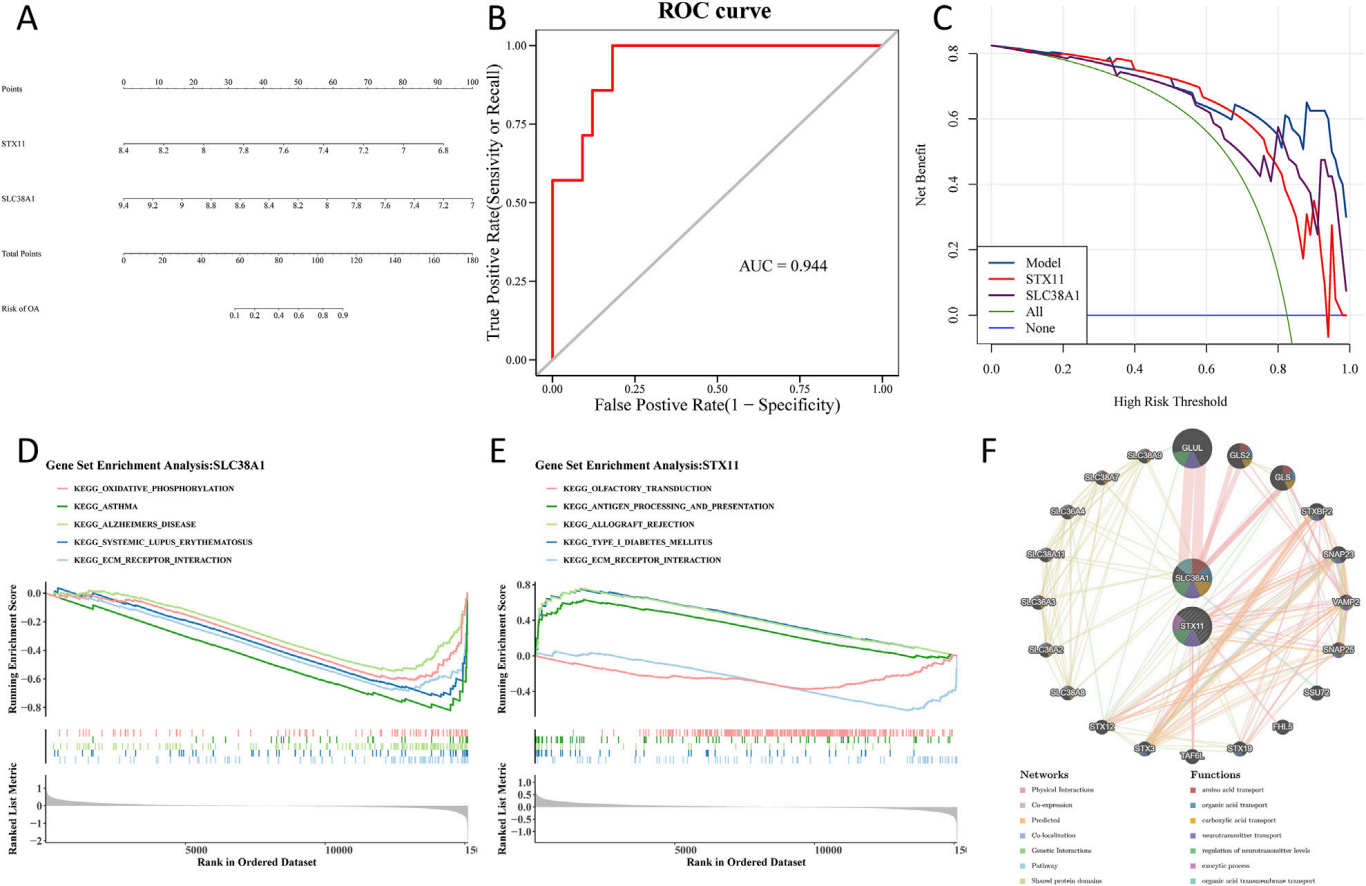
Construction of a nomogram and functional analysis of hub genes. **(A)** A nomogram is used to predict the risk of OA. **(B)** Area under the curve value of the ROC curve. **(C)** Model evaluation curves show that the model containing the two identified hub genes has a higher net benefit. **(D)** Gene set enrichment analysis of the *SLC38A1* gene. **(E)** Gene set enrichment analysis of the *STX11* gene. **(F)** GGI network using GeneMANIA.

Based on the hub genes, a nomogram was constructed to predict the risk of OA (Figure 6A). The area under the curve (AUC) value of the ROC curve was 0.944, which demonstrated that the nomogram had a good ability to diagnose OA and control samples (Figure 6B). Furthermore, DCA showed that the nomogram and hub genes had a higher net benefit than the extreme curve and single factor (Figure 6C). To find biological functions involved in hub genes, GSEA was performed. The results showed that the ECM–receptor interaction was consistently among the top five enriched pathways based on enrichment scores of the hub genes (Figures 6D, E). Through GeneMANIA, we identified 20 genes that are functionally related to hub genes. A GGI network was constructed, showing interactions such as *GLUL*–*SLC38A1*, *GLS2*–*SLC38A1*, and *GLS*–*SLC38A1*. This analysis revealed that hub genes were involved in multiple vital biological processes, including amino acid transport, carboxylic acid transport, and regulation of neurotransmitter levels (Figure 6F).

### 3.6 Hub genes correlate with NK cell infiltration in OA cartilage

**FIGURE 7:**
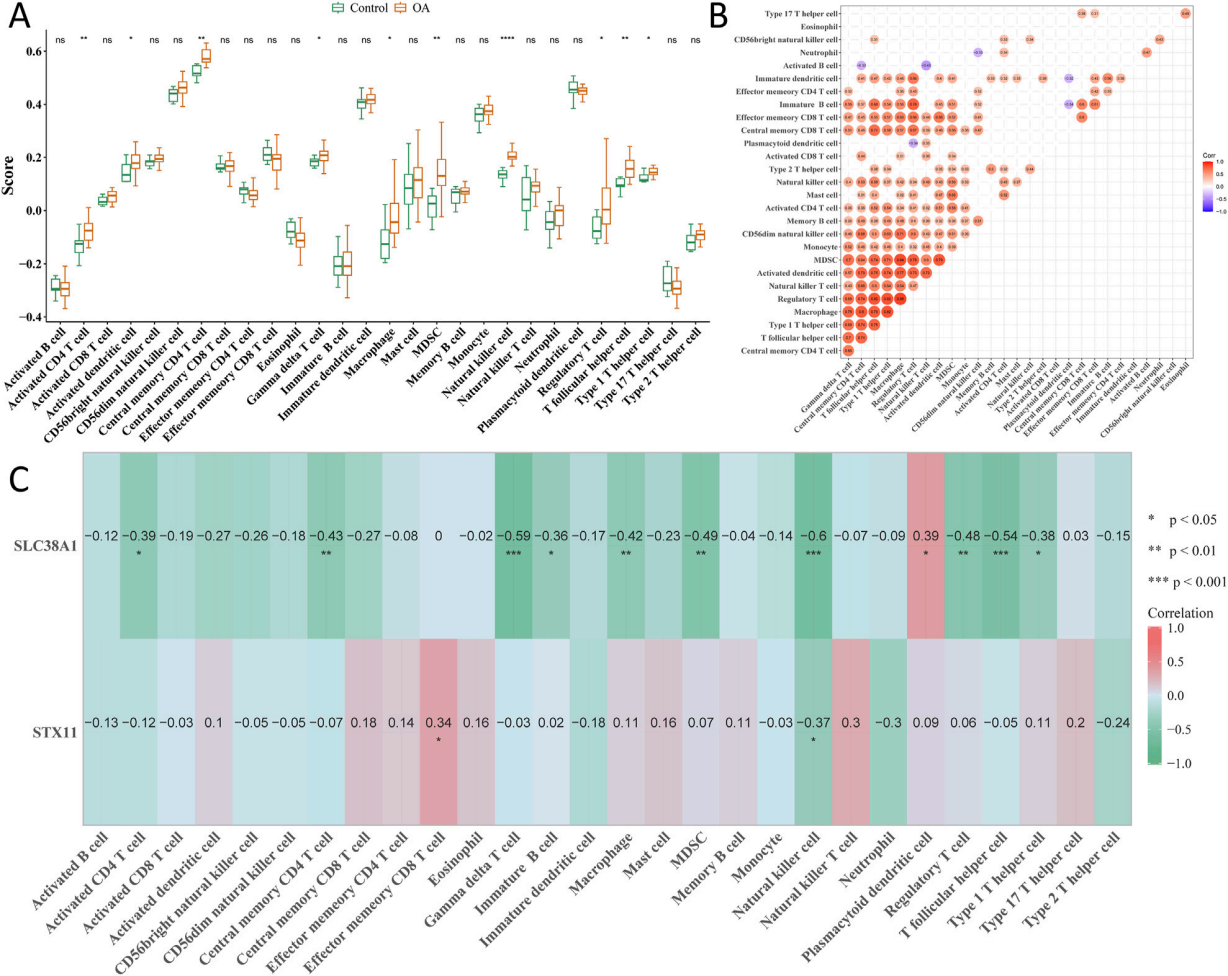
Analysis of immune cell components in single-cell sequencing. **(A)** Cell fraction difference of 28 immune cell types between the OA and control groups. **(B)** Correlation analysis of all immune cells. **(C)** Correlation between hub genes and all immune cells. *p < 0.05, **p < 0.01, and ***p < 0.001.

Among 28 immune cell types, 10 types of immune cells had significant differences between the OA and control groups, including activated CD4 T cells, activated dendritic cells, and central memory CD4 T cells (Figure 7A). Correlation analysis showed that activated B cells had the strongest negative correlation with natural killer (NK) T cells (cor = −0.43; *p* < 0.05), while regulatory T cells had the strongest positive correlation with macrophages (cor = 0.88; *p* < 0.05) (Figure 7B). Moreover, hub genes had a negative correlation with NK cells (cor < −0.3; *p* < 0.05), and *SLC38A1* was positively correlated with plasmacytoid dendritic cells (cor >0.3; *p* < 0.05) (Figure 7C).

### 3.7 Comprehensive regulatory network and drug prediction of hub genes

**FIGURE 8:**
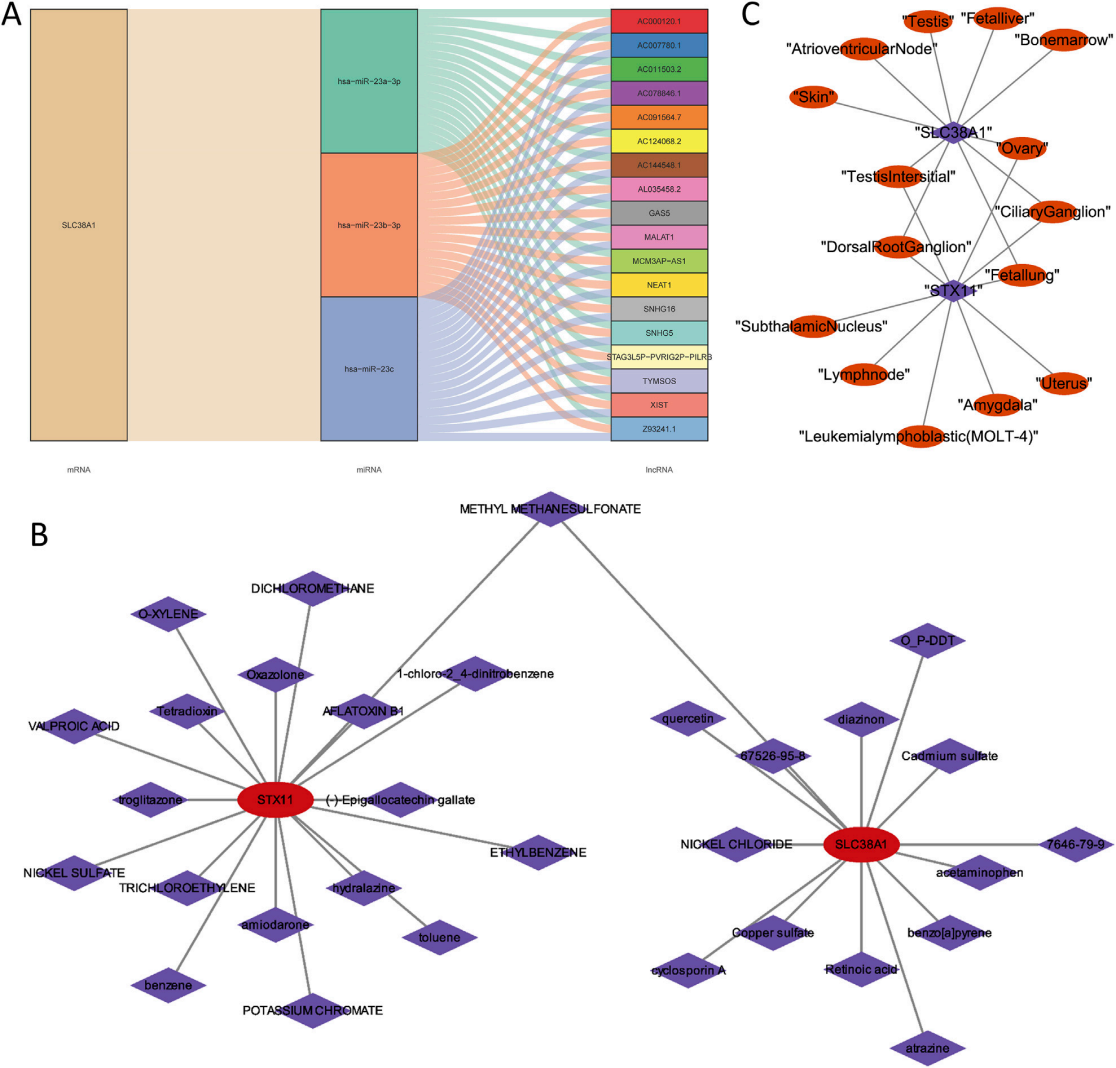
Analysis of the lncRNA–miRNA–mRNA regulatory network and drugs prediction of two hub genes. **(A)** LncRNA–miRNA–mRNA regulatory network of *SLC38A1*. **(B)** Predicted drugs for *SLC38A1* and *STX11*. **(C)** Top 10 tissues with the highest expression scores for the two hub genes.

To understand the regulatory relationship of hub genes, an lncRNA–miRNA–mRNA regulatory network was constructed with *SLC38A1*, 3 miRNAs, and 18 lncRNAs. In this network, all lncRNAs regulated *SLC38A1* through hsa-miR-23a-3p, hsa-miR-23b-3p, and hsa-miR-23c (Figure 8A). Through computer simulation assumptions, *SLC38A1* predicted a total of 14 drugs, and *STX11* predicted a total of 18 drugs. Among these drugs, methyl methanesulfonate was predicted by *SLC38A1* and *STX11*, quercetin was predicted by *SLC38A1*, and oxazolone was predicted by *STX11* (Figure 8B). Furthermore, the top 10 tissues with the highest expression scores for the two hub genes commonly included the testis interstitium, ovary, dorsal root ganglion, ciliary ganglion, and fetal lung (Figure 8C).

### 3.8 Endothelial cells, monocytes, and T cells were identified as key cells

**FIGURE 9:**
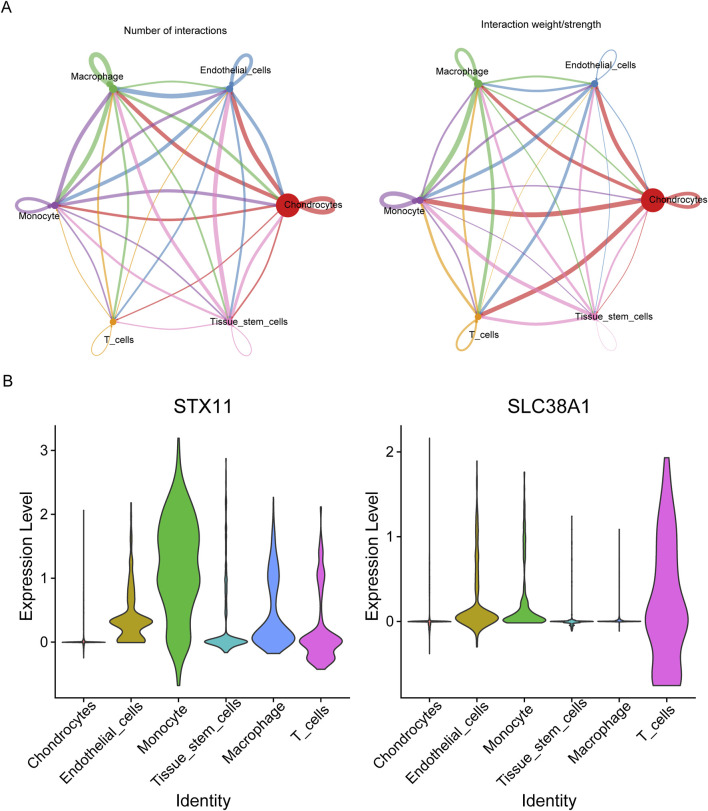
Communication between different cell types. **(A)** Interaction number and strength between different cell clusters. **(B)** Violin plots of the expression levels of two hub genes in various cell clusters.

Through cell communication analysis, chondrocytes were shown to have a strong correlation with other cell subpopulations (Figure 9A). Following that, monocytes had the strongest correlation with monocytes, and the ligand–receptor pairs were *IL-1B*–*IL-1R2* (Supplementary Figure S3). To select key cells, the expression of hub genes was analyzed, and the results showed that hub genes exhibited higher and widespread expression in endothelial cells, monocytes, and T cells. Thus, these three cell types were identified as key cells for subsequent analysis (Figure 9B). There were five stages of differentiation for endothelial cells, arranged from top to bottom (Supplementary Figure S4A). For monocytes and T cells, there were eight and three differentiation stages, respectively, arranged from left to right (Supplementary Figure S4B, C). In endothelial cells, the expression of *SLC38A1* initially increased in early differentiation stages and then decreased before gradually increasing again. In contrast, *STX11* showed a gradual decrease initially, followed by a slight increase and a subsequent rapid increase during differentiation. For monocytes, *SLC38A1* expression increased gradually throughout differentiation, while *STX11* initially increased slowly, followed by a plateau phase. In T cells, *SLC38A1* expression initially increased and then declined rapidly, whereas *STX11* showed a slow initial decrease, followed by a gradual increase and a subsequent decline during differentiation (Supplementary Figure S5). According to scRNA-seq data, 195 TFs were screened out, and the top 30 TFs included *TBX21*, *SPIB*, and *MAF* (Supplementary Figure S4D).

### 3.9 Differential expression of *SLC38A1* in the OA and control groups

**FIGURE 10:**
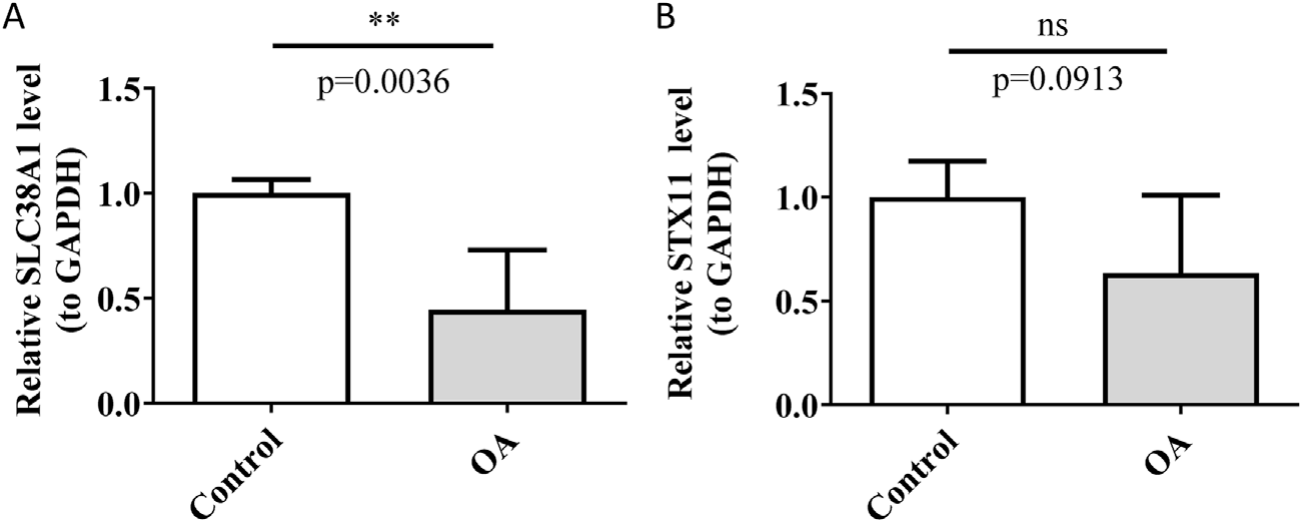
Detection of relative expression levels of *SLC38A1* and *STX11*. **(A)** Relative expression level of *SLC38A1* in OA and control tissues. **(B)** Relative expression level of *STX11* in OA and control tissues. **p < 0.01; ns represents no statistical significance.

RT-qPCR is a molecular biology technique that is widely used in clinical diagnostics and drug development to measure the expression levels of specific genes in samples (Falini and Dillon, 2024). In this study, RT-qPCR was utilized to assess the mRNA expression levels of hub genes to investigate their potential differential expression in OA samples. The results showed differential expression of *SLC38A1* between the control and OA groups, with lower expression levels detected in the OA group (*p* < 0.01) (Figure 10A). The result consisted of the expression trend of *SLC38A1* in the OA group in the GSE57218 and GSE117999 datasets. However, the expression level of *STX11* in the OA group exhibited a decrease, but the change did not reach statistical significance (*p* > 0.05) (Figure 10B). This might be due to the small sample size used, and it will need to be confirmed with a larger sample size in future studies.

## 4 Discussion

In this study, we aimed to unravel the intricate mechanisms linking MD-RGs and MP-RGs in OA. The findings significantly contribute to the understanding of the pathogenesis of OA at the molecular level and offer new perspectives for its diagnosis and treatment.

The pathways enriched by candidate genes—such as phagocytic vesicle membrane, type-I diabetes mellitus, and cell adhesion molecules—have been previously reported in the literature to be associated with OA (Rios-Arce et al., 2022; Njoto et al., 2019; Cheng et al., 2020; Chen et al., 2020). According to research, TMEM230 promotes antigen processing, transport, and presentation by regulating the membrane-bound organelles. It also plays a crucial role in regulating the secretion of metalloproteinase and heparanase, which are required for tissue remodeling in OA and rheumatoid arthritis (RA) (Abeni et al., 2024). Type-I diabetes mellitus may suppress the development of post-traumatic osteoarthritis (Rios-Arce et al., 2022). Neural cell adhesion molecule (NCAM) could inhibit hypertrophic chondrocyte differentiation of mesenchymal stem cells by suppressing ERK signaling, thereby reducing chondrocyte hypertrophy in OA models. These findings suggest that the potential utility of NCAM as a novel therapeutic target for OA (Cheng et al., 2020).

We found that *SLC38A1* was downregulated and that *STX11* showed a downregulated expression trend in OA, which might serve as potential biomarkers associated with mitochondrial dynamics and mitophagy in OA through bioinformatics analysis. *SLC38A1* (solute carrier family 38, member 1) is a sodium-coupled neutral amino acid transporter, which is primarily responsible for the transport of amino acids across the cell membrane. Although there are currently no reports on the functional mechanism of the *SLC38A1* gene in osteoarthritis, many other members of the solute carrier family have been reported to play a role in OA. For example, capsiate inhibited the expression of *HIF-1α* by activating *SLC2A1*, thereby reducing the progression of ferroptosis-related osteoarthritis (Guan et al., 2023). Additionally, miR-19b-3p in exosomes from osteoarthritic fibroblast-like synoviocytes enhanced chondrocyte ferroptosis and damage in OA by sponging off *SLC7A11* (Kong et al., 2023). *STX11* (Syntaxin 11) is mainly expressed in immune cells, is involved in cytotoxic granule exocytosis, and is crucial for the cytotoxic functions of CD8^+^ T cells and NK cells (D'Orlando et al., 2013). In a bioinformatics study of rheumatoid arthritis and osteoarthritis, *STX11* was found to be highly enriched in protein–protein interaction networks (Qiu et al., 2021). Additionally, *STX11* may modulate OA-associated inflammation and OA progression by affecting the activity of macrophages, T cells, and NK cells (D'Orlando et al., 2013; Offenhäuser et al., 2011; Dabrazhynetskaya et al., 2012). Thus, *STX11* may be vital in OA pathogenesis via immune cell function and cytokine regulation, further suggesting its potential relevance in OA and RA. Future research should use knockdown or overexpression techniques in cell models and gene agonists or inhibitors in animal models to further clarify the specific molecular mechanisms of *SLC38A1* and *STX11* in OA progression.

Single-cell analysis showed high *SLC38A1* and *STX11* expression in monocytes and immune cells, and the proportion of NK cells was found to be significantly higher in the OA group than in the control group, according to immune infiltration analysis. Monocytes, when exposed to various stimuli, express ligands for the immune receptor found on NK cells. These ligands activate NK cells and enhance their cytotoxicity (Getz et al., 2007). Additionally, both monocytes and NK cells secrete cytokines that can influence each other’s functions. Monocytes produce *IL-15*, which boosts NK cells’ cytotoxicity and *IFN-γ* production (Cooper et al., 2001). In turn, *IFN-γ* from NK cells activates monocytes and enhances their antigen presentation ability (Cooper et al., 2001). These immune cells’ interactions may influence OA progression. Moreover, the correlation analysis of *SLC38A1* and *STX11* with all immune cells showed a negative correlation with NK cells, further suggesting that *SLC38A1* and *STX11* may influence the development of osteoarthritis through the immune infiltration of NK cells.

The construction of the lncRNA–-miRNA–mRNA regulatory network for *SLC38A1* provided insights into the post-transcriptional regulation of this hub gene. The prediction of drugs associated with *SLC38A1* and *STX11*, such as methyl methanesulfonate, quercetin, and oxazolone, offers potential therapeutic targets for OA treatment. Quercetin alleviates the progression of osteoarthritis by regulating inflammatory cascades and chondrocyte apoptosis (Hu et al., 2019; Wang et al., 2023), and *SLC38A1* may serve as an intermediate through which it exerts these effects. These predicted drugs could potentially modulate the functions of the hub genes and, thus, alleviate the pathological symptoms of OA.

However, there are limitations to this study, including limited *in vitro* and *in vivo* validation of the functions of *SLC38A1* and *STX11*, the use of RT-qPCR to validate the expression of hub genes, and the need for more versatile experiments, such as gene knockout or overexpression in cell or animal models, to fully understand their role in OA. Second, the small sample size of RT-qPCR and scRNA-seq data, along with the lack of control samples in the scRNA-seq dataset, may affect the accuracy of the result. Only *SLC38A1* showed significant differential expression in RT-qPCR. In addition, *STX11* did not reach statistical significance, which might be due to the small sample size. However, the *STX11* expression trend matched the bioinformatics results in the GSE57218 and GSE117999 datasets, and it was widely expressed in key cell subsets such as endothelial and mononuclear cells in single-cell data, leading us to designate it as a hub gene. Additionally, while the study primarily focused on the relationship between mitochondrial-related genes and OA, other contributing factors were not fully explored. Moreover, the lncRNA–miRNA–mRNA regulatory network and drug predictions were based on database predictions. The actual regulatory roles need further exploration of their molecular mechanisms through molecular experiments (such as siRNA and mimic) and cellular models (such as drug induction). Future studies should expand the scope to deepen our understanding of OA and develop more comprehensive diagnostic and therapeutic strategies.

## Data Availability

The original contributions presented in the study are included in the article/Supplementary Material, further inquiries can be directed to the corresponding authors.
