# Supplementary figures and images for: SLC38A1 and STX11 are mitochondria-related biomarkers associated with immune infiltration in osteoarthritis
Source: Front Genet. 2025 Jul 30;16:1585775. doi: 10.3389/fgene.2025.1585775 (PMC12343263; doi:10.3389/fgene.2025.1585775)

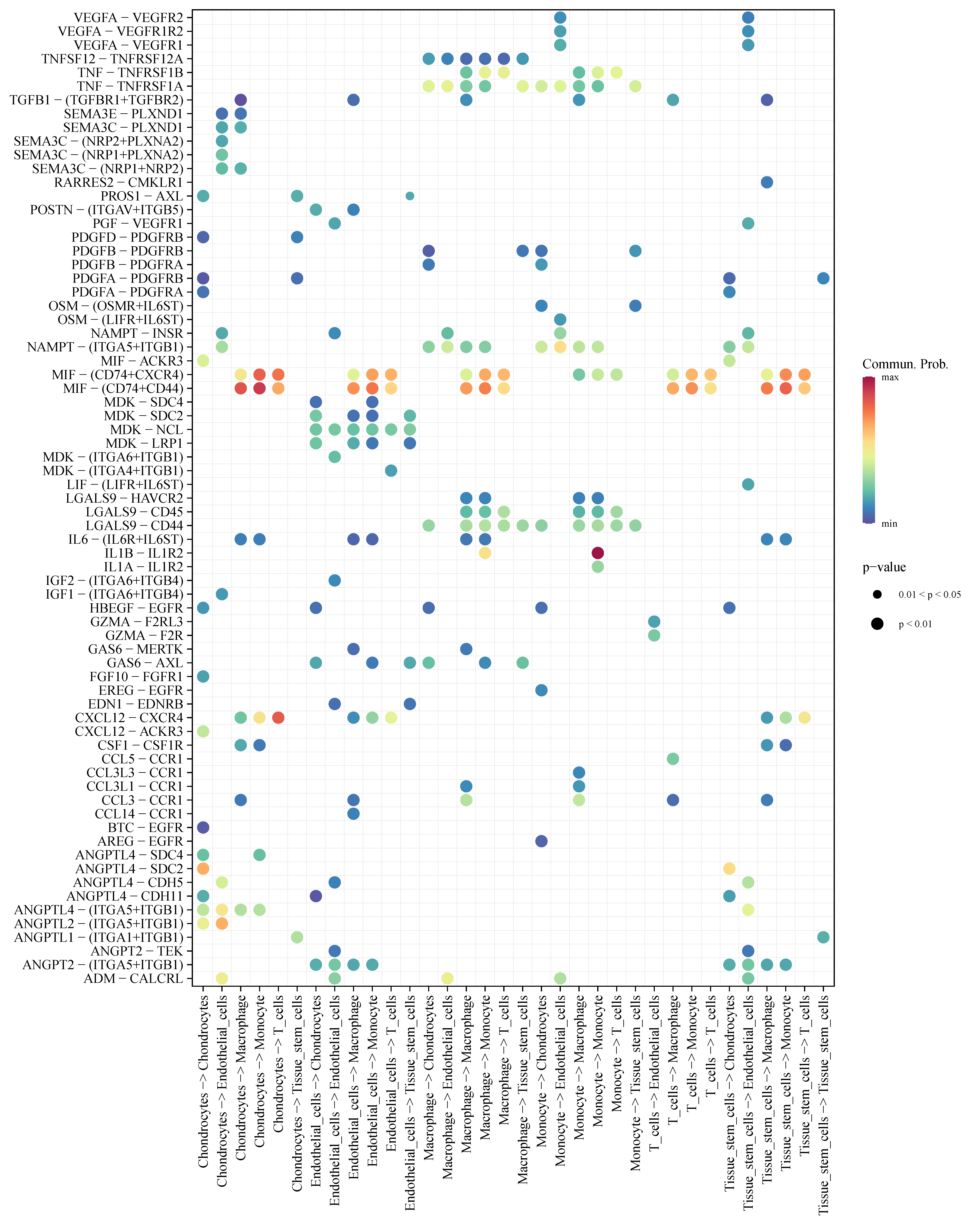

Supplement: Supplementary file 1 [file Image3.tiff]

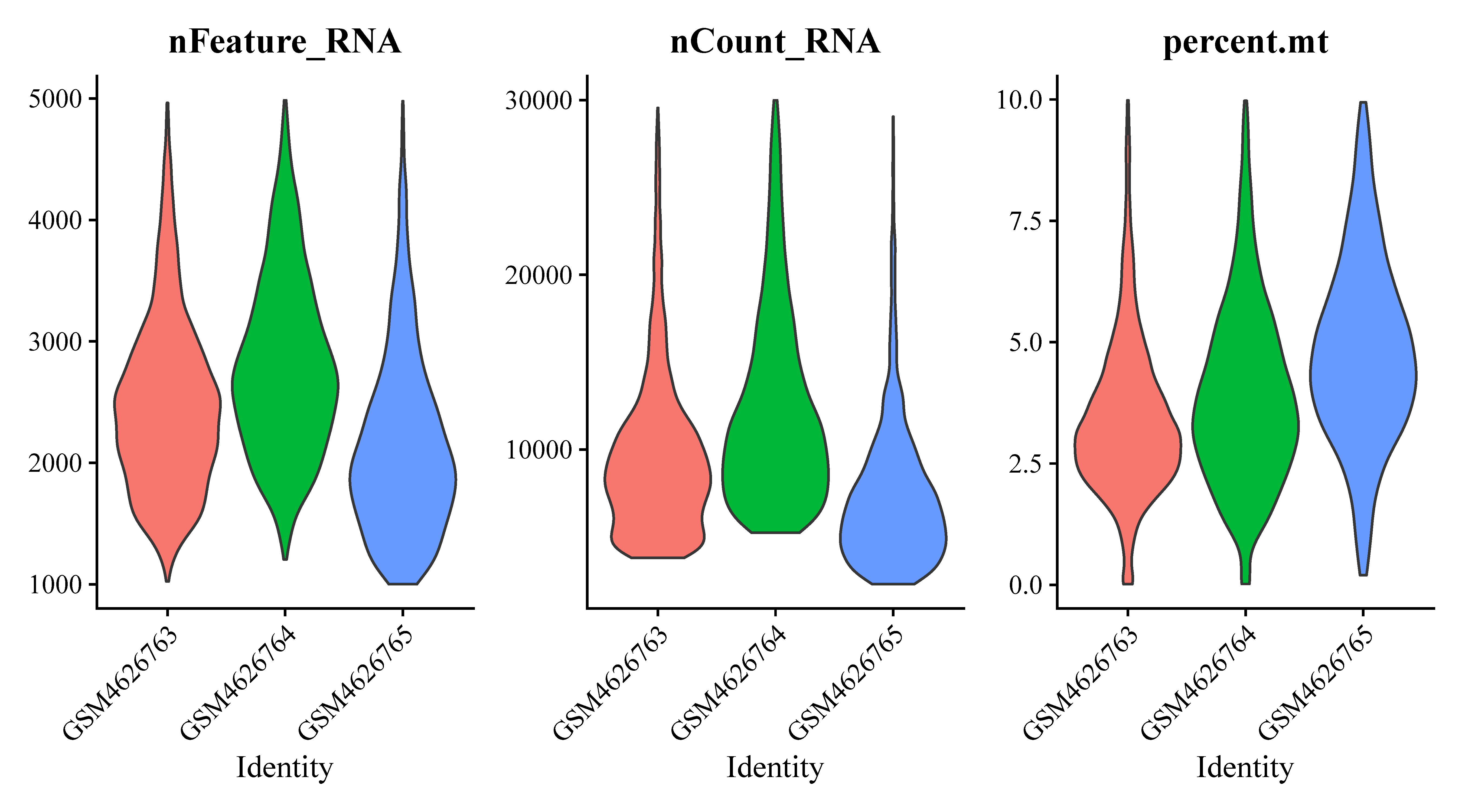

Supplement: Supplementary file 2 [file Image1.tiff]

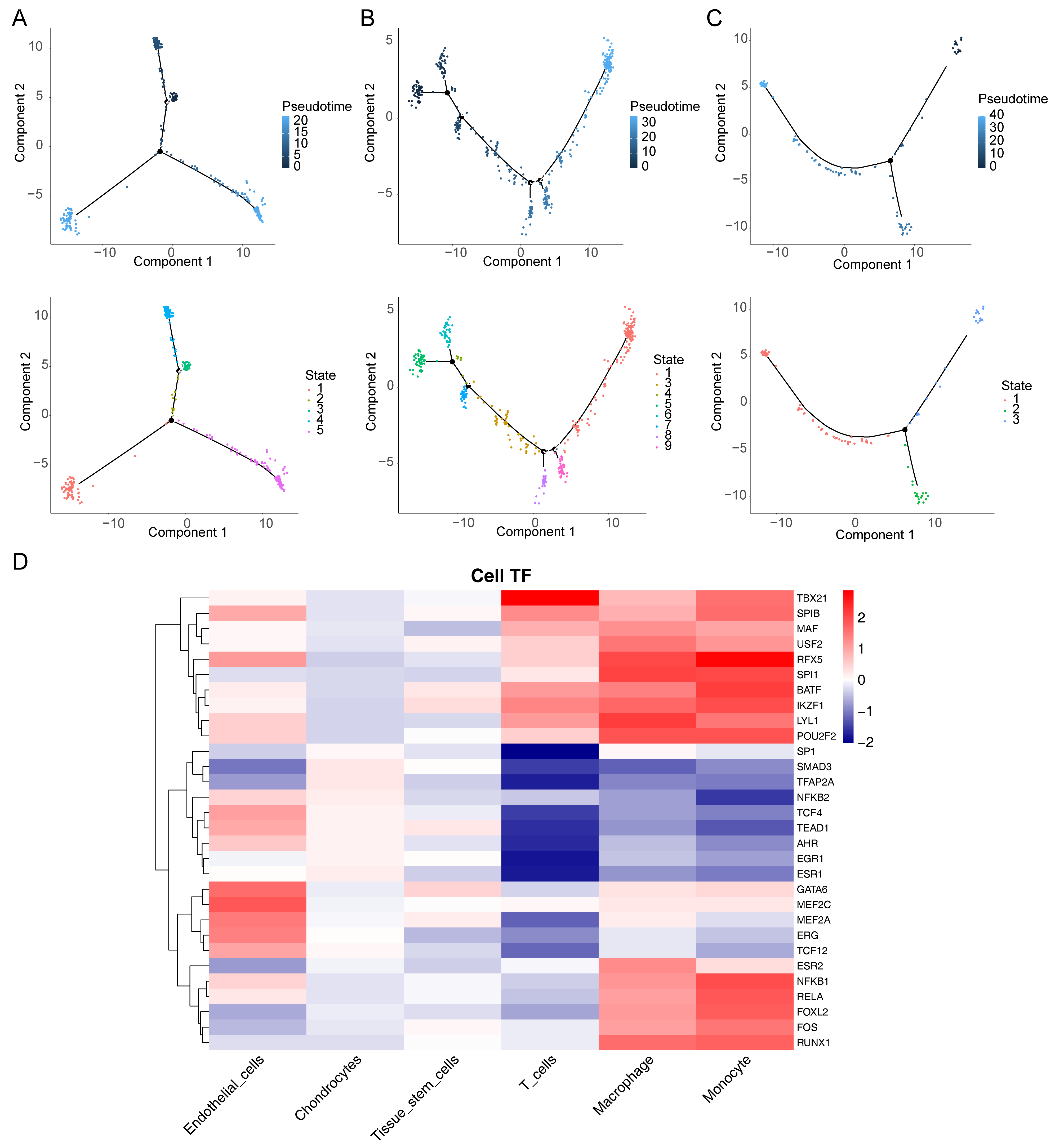

Supplement: Supplementary file 3 [file Image4.tif]

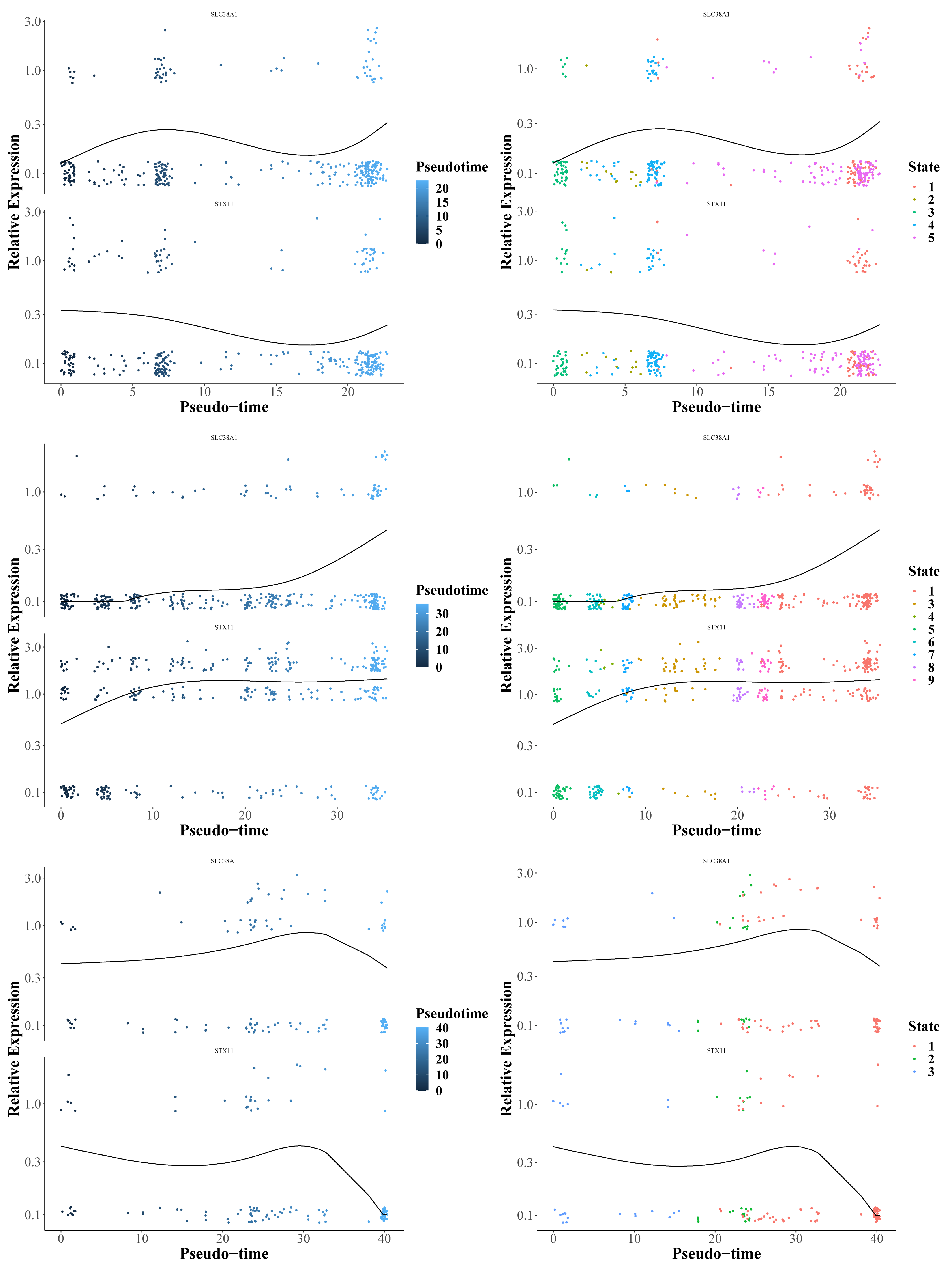

Supplement: Supplementary file 7 [file Image5.tif]

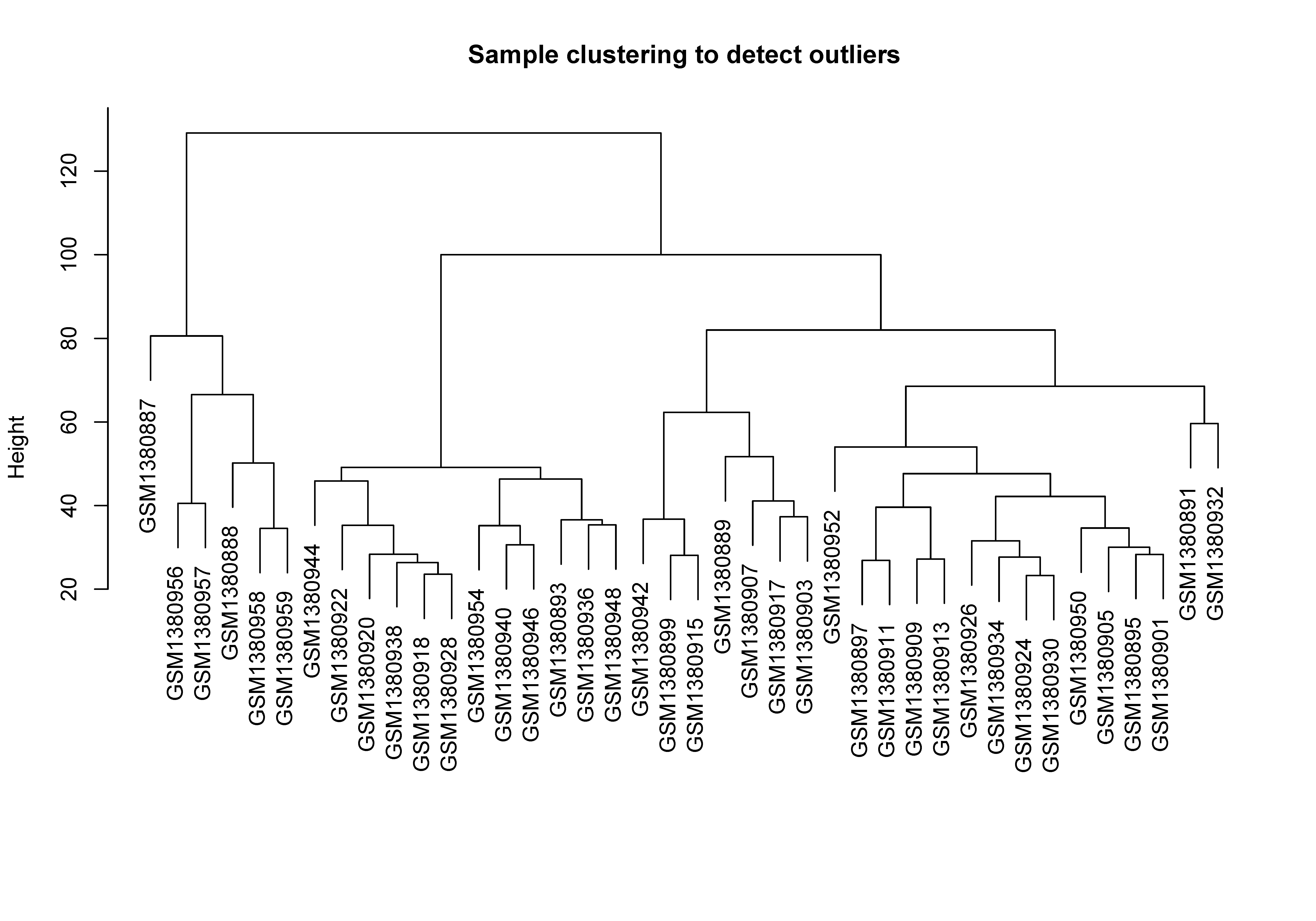

Supplement: Supplementary file 8 [file Image2.tiff]
