# Supplementary material for: SLC38A1 and STX11 are mitochondria-related biomarkers associated with immune infiltration in osteoarthritis
Source: Front Genet. 2025 Jul 30;16:1585775. doi: 10.3389/fgene.2025.1585775 (PMC12343263; doi:10.3389/fgene.2025.1585775)
Supplement: Supplementary file 9 [file Table2.doc]

**Table S1 The Primer Sequences of hub genes**

| Primer | Sequences |
| --- | --- |
| SLC38A1 F | CCAGCGGAAGGAACAGGAAG |
| SLC38A1 R | ATGGAAGCTTGACACCCCTG |
| STX11 F | CTCCCAGTCCAGGCAAAATG |
| STX11 R | GTCAAACTCATCGTCCCCGT |
| internal reference -GAPDH F | CGAAGGTGGAGTCAACGGATTT |
| internal reference -GAPDH R | ATGGGTGGAATCATATTGGAAC |
